# Case Report: Acute myocarditis in a patient with Duchenne muscular dystrophy

**DOI:** 10.3389/fcvm.2024.1419496

**Published:** 2024-09-03

**Authors:** Xinyuan Zhang, Yingkun Guo, Huayan Xu

**Affiliations:** Department of Radiology, Key Laboratory of Birth Defects and Related Diseases of Women and Children, Ministry of Education, West China Second University Hospital, Sichuan University, Chengdu, China

**Keywords:** Duchenne muscular dystrophy, acute myocarditis, cardiac troponin I, cardiac magnetic resonance, case report

## Abstract

**Background:**

Cardiovascular complications are the leading cause of death among individuals with Duchenne muscular dystrophy (DMD). However, due to the difficulty in evaluating individuals with inactive DMD, acute myocardial injury may be overlooked.

**Case presentation:**

An 11-year-old boy with DMD presented to the emergency department with a 5-day history of persistent nasal congestion, runny nose, and cough. He was regularly taking prednisolone acetate, angiotensin-converting enzyme (ACE) inhibitors, and β-blockers for suspected DMD-associated cardiomyopathy. Upon presentation, a substantially elevated cardiac troponin I (cTnI) level of 19.8 μg/L and abnormal electrocardiogram (ECG) results were detected. Further cardiac magnetic resonance imaging (CMR) showed myocardial inflammation with localized T2 hyperintensity from the basal to middle lateral and inferior walls, as well as late gadolinium enhancement (LGE) from the basal to apical inferior lateral walls, supporting a diagnosis of acute myocarditis. Subsequently, the patient showed clinical improvement in response to combination treatment with intravenous immunoglobulin, oral prednisolone acetate, potassium chloride sustained-release tablets, anti-heart failure medication, and broad-spectrum antibiotics.

**Conclusions:**

We report a rare case of acute myocarditis in a patient with DMD, potentially due to upper respiratory tract infection. This case highlights the importance of early myocarditis recognition and treatment in patients with DMD.

## 1 Introduction

Duchenne muscular dystrophy (DMD) is an X-linked genetic disorder caused by mutations in the dystrophin gene, with an incidence of approximately 1 in 5,000 male births (1). The pathological features of DMD include muscle degeneration, necrosis, inflammation, edema, and subsequent fibrous tissue deposition, leading to progressive skeletal and cardiac muscle disease (2, 3). Cardiovascular complications are the leading cause of death among individuals with DMD (4). Dystrophin deficiency in the heart manifests as cardiomyopathy. Early diagnosis and appropriate treatment of cardiomyopathy in patients with DMD are crucial for efforts to improve prognosis.

There is some evidence that chronic myocardial injury may be mitigated by the initiation of angiotensin-converting enzyme (ACE) inhibitors and β-blockers in asymptomatic boys with DMD who exhibit normal left ventricular systolic function around age 10 (4–7). However, acute myocardial injury may be overlooked in individuals with inactive DMD due to the difficulty in evaluating their signs and symptoms.

Here, we report a rare case of acute myocarditis due to upper respiratory tract infection in an 11-year-old boy with DMD who was regularly receiving ACE inhibitors and β-blockers. This case may provide new insights into the pathogenesis, diagnosis, and management of DMD-associated cardiomyopathy complicated by acute myocarditis.

## 2 Case description

**Figure 1:**
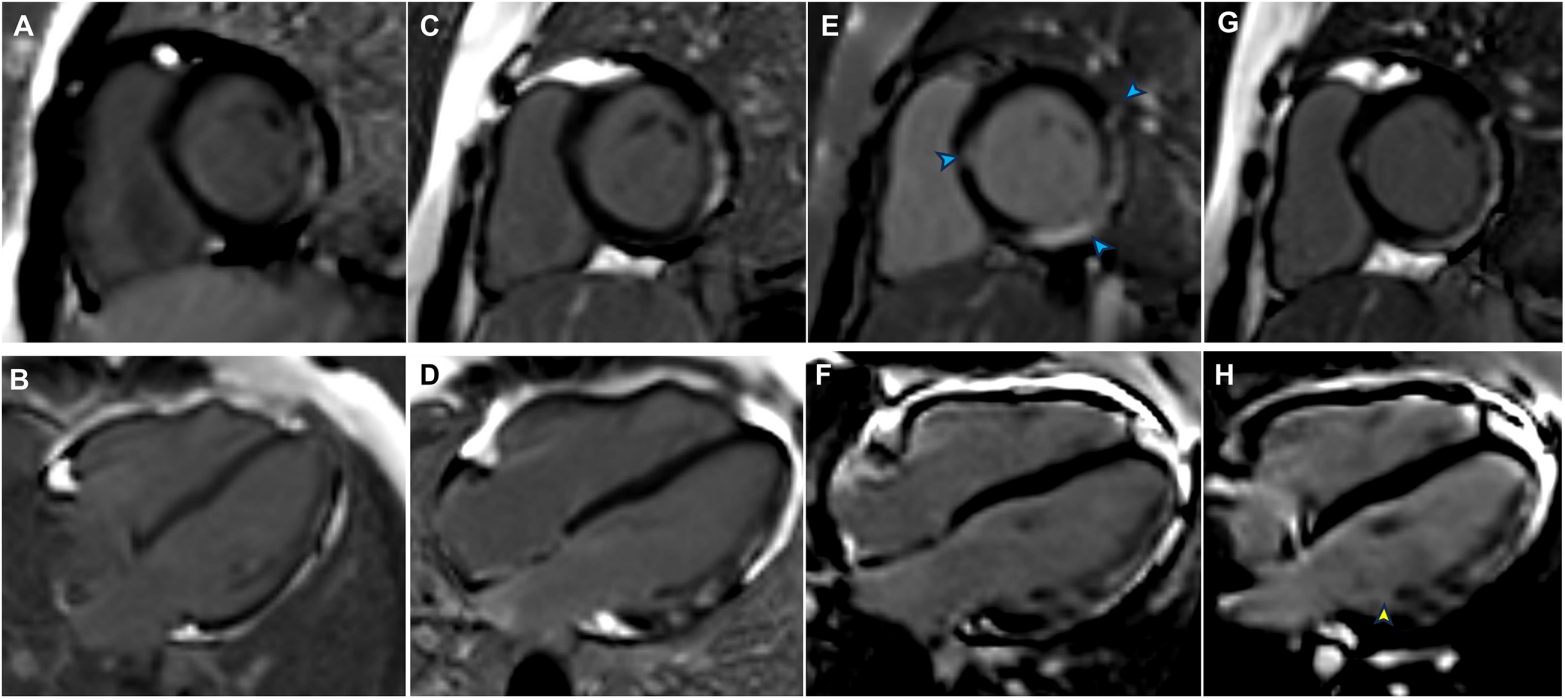
Late gadolinium enhancement (LGE) findings from previous follow-up, admission, and post-discharge follow-up. **(A)** LGE of the left ventricular (LV) lateral wall in August 2020 (LV short-axis views). **(B)** LGE of the LV lateral wall in August 2020 (LV long-axis views). **(C)** LGE of the LV lateral wall from the basal to middle segments in January 2023 (LV short-axis views). **(D)** LGE of the LV lateral wall from the basal to middle segments in January 2023 (LV long-axis views). **(E)** LGE was present in the interventricular septum and inferolateral lateral walls (blue arrowheads) at admission (LV short-axis views). **(F)** Progression of LGE in the inferolateral walls at admission (LV long-axis views). **(G)** LGE persisted in the interventricular septum and inferolateral lateral walls in July 2024 (LV short-axis views). **(H)** An increased extent of LGE was evident in the inferolateral walls (yellow arrowhead) in July 2024 (LV long-axis views).

An 11-year-old boy with DMD presented to the emergency department with a 5-day history of persistent nasal congestion, runny nose, and cough. He denied fever, chest pain, or palpitations. The patient had been diagnosed with DMD at the age of 5 due to the deletion of exons 53 and 54 in the dystrophin gene. He had begun taking prednisolone acetate (25 mg daily) and regularly attended outpatient follow-up. At the time of presentation to our hospital, the patient had a 3-year history of suspected DMD-associated cardiomyopathy, based on the presence of late gadolinium enhancement (LGE) in the left ventricular lateral wall on screening cardiac magnetic resonance imaging (CMR) (Figures 1A–D). The patient exhibited positive responses to ACE inhibitors (10 mg enalapril daily) and β-blockers (50 mg metoprolol daily), which he had been taking prior to admission.

**Figure 2:**
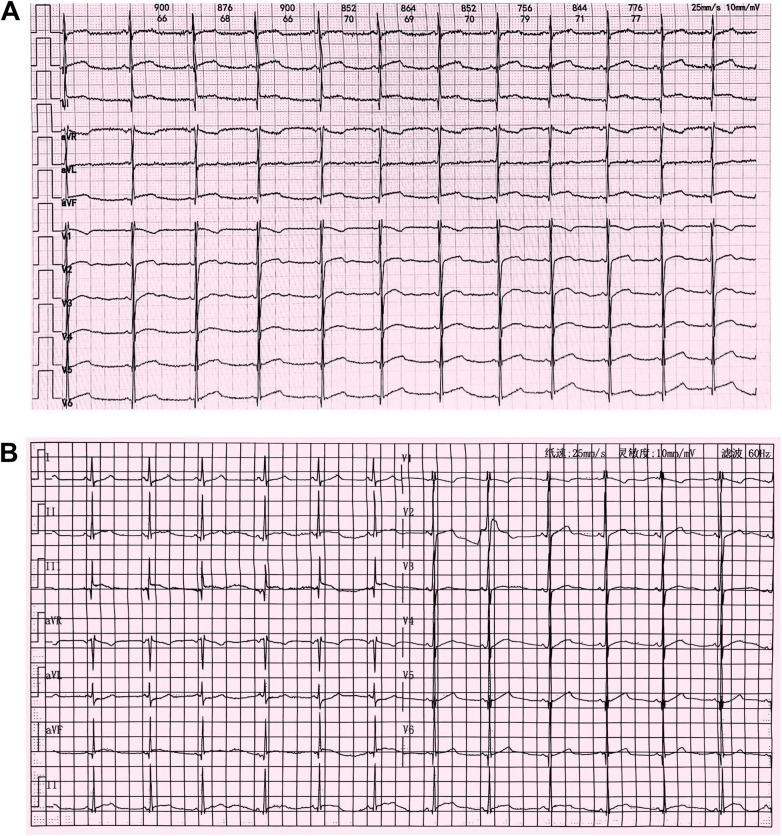
**(A)** Electrocardiogram at admission in October 2023 showing mild ST elevations and an abnormal Q wave in leads II, III, and aVF. **(B)** Follow-up electrocardiogram after improvement of myocarditis in February 2024 showing abnormal Q waves and T-wave flattening or inversion in leads II, III, and aVF.

Upon presentation to our hospital, the patient's vital signs were as follows: temperature, 36.6°C; blood pressure, 105/67 mmHg; heart rate, 90 bpm; respiratory rate, 22 breaths/min; and oxygen saturation, 98% on room air. Heart and lung auscultation findings were normal. Laboratory tests revealed the following: white blood cell count, 16,630/μl (76.5% neutrophils); C-reactive protein, 7.8 mg/L; serum CK-MB, 277.0 ng/ml (reference range 0.0–25.0 ng/ml); and myoglobin, 1,025.4 μg/L (reference range 0.0–110.0 μg/L). A substantially elevated cardiac troponin I (cTnI) level of 19.8 μg/L (reference range 0.0–0.06 μg/L) was detected. There was no evidence of infection, as indicated by negative IgM serology results for Epstein-Barr virus (EBV), respiratory syncytial virus (RSV), coxsackievirus, adenovirus, *Mycoplasma pneumoniae*, and *Chlamydia pneumoniae*. Chest x-rays showed a normal cardiac silhouette without cardiomegaly or pulmonary edema. An electrocardiogram (ECG) revealed slight ST elevation and abnormal Q waves in the inferior wall (Figure 2A). An echocardiogram indicated normal heart morphology without valvular disease or segmental wall motion abnormalities; the left ventricular ejection fraction (LVEF) was 56%.

**Figure 3:**
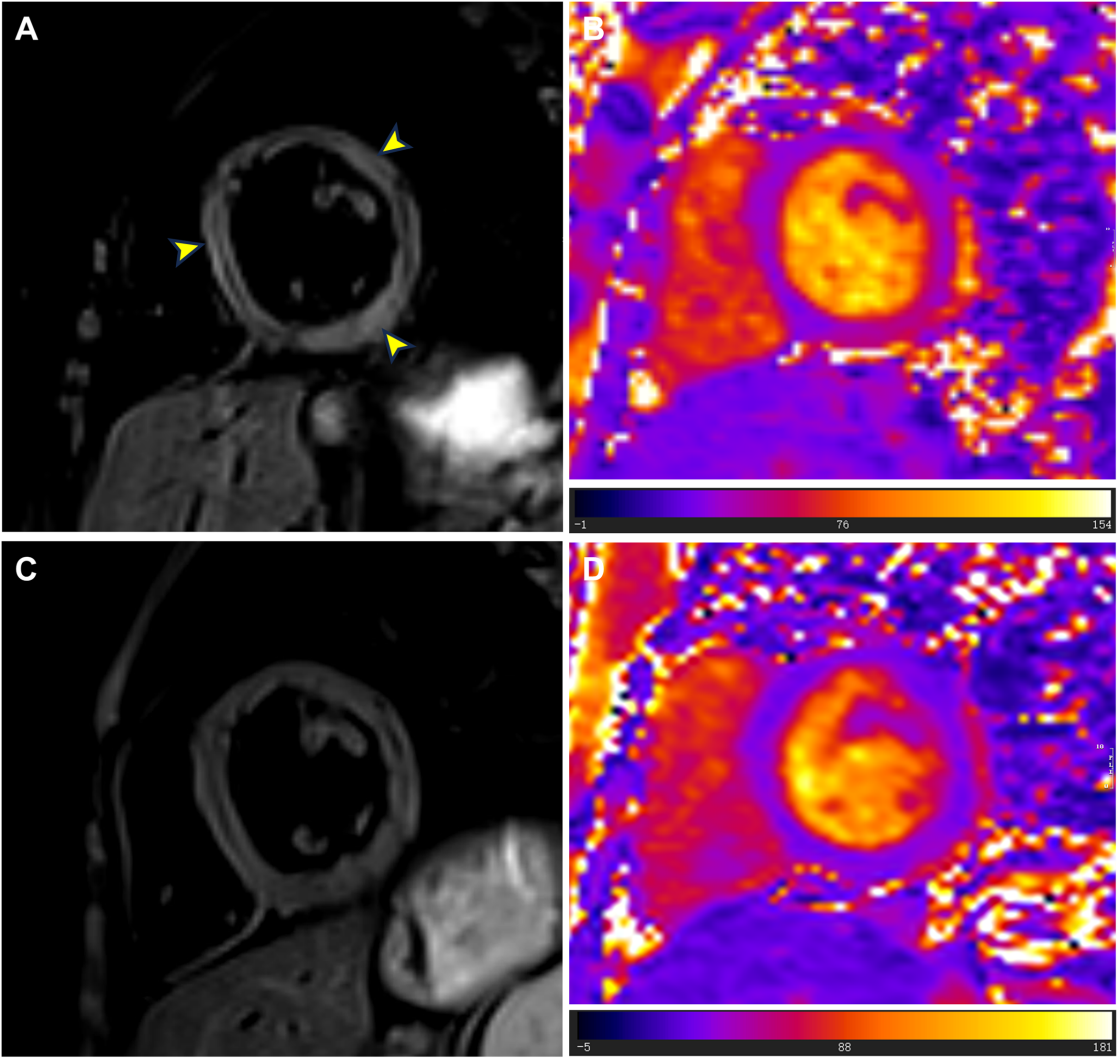
T2-weighted imaging and T2 mapping images at admission and follow-up. **(A)** At admission, T2-weighted imaging showed localized high signals in the left ventricular septum, lateral wall, and inferior wall (yellow arrowheads). **(B)** At admission, T2 mapping images showed abnormally elevated T2 values (mean value = 56.9 ms) in the basal to middle segments of the interventricular septum and inferolateral walls. **(C)** During the follow-up cardiac magnetic resonance examination in July 2024, T2-weighted imaging showed no signs of myocardial edema. **(D)** During the follow-up cardiac magnetic resonance examination in July 2024, T2 mapping images showed reduced T2 values (mean value = 42.0 ms) in the basal to middle septal and inferolateral walls.

Although the patient did not report any cardiac symptoms, the elevated cTnI levels and abnormal ECG findings indicated possible myocardial necrosis. Thus, a CMR examination was recommended. CMR showed inflammatory myocardial edema with localized T2 hyperintensity (T2 ratio, 2.4; T2 mapping, 56.9 ± 0.4 ms) in the inferolateral walls and interventricular septum from the basal to middle segments (Figures 3A,B). Additionally, LGE progression in the inferolateral walls and interventricular septum was observed, supporting a diagnosis of acute myocarditis (Figures 1E,F).

**Figure 4:**
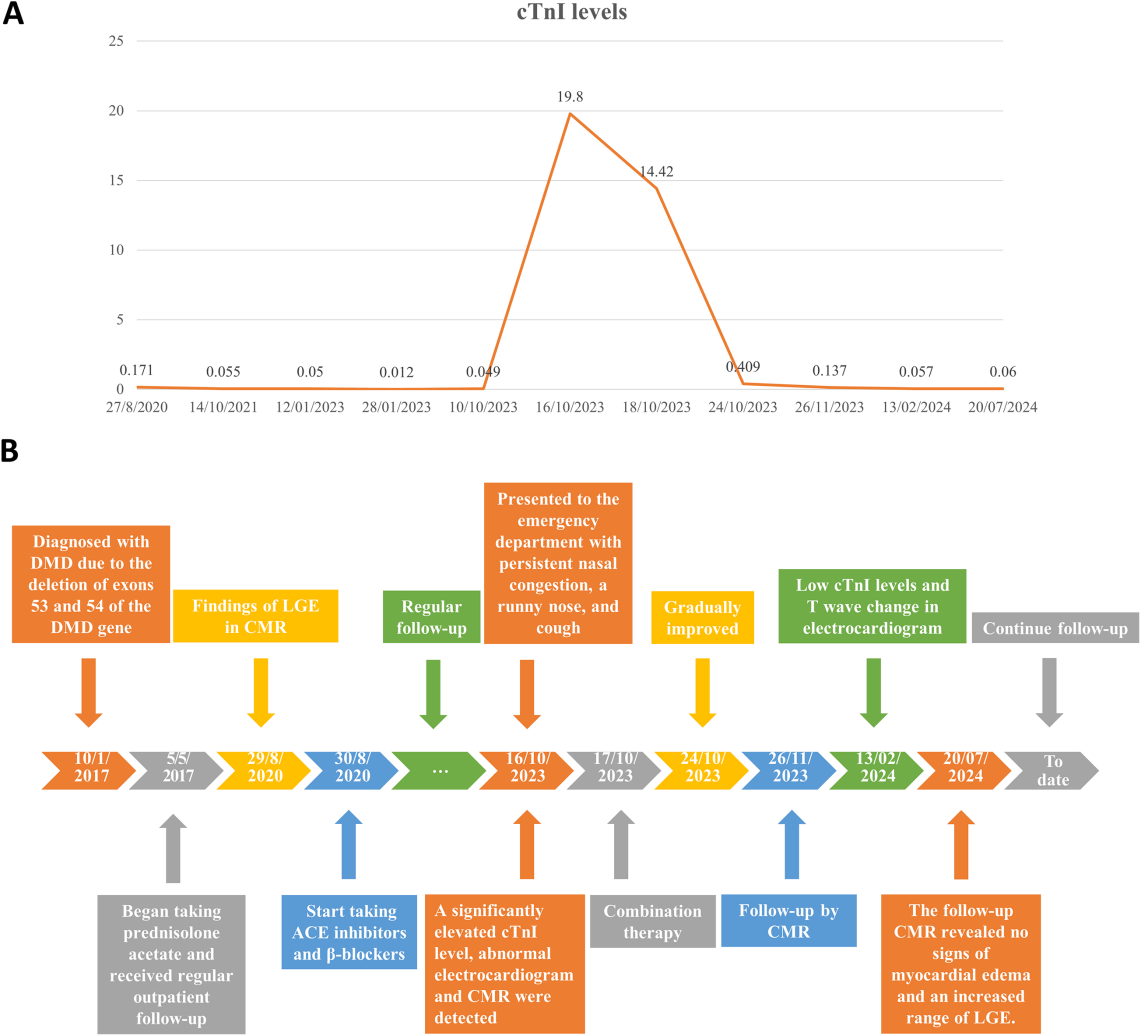
**(A)** Changes in the patient's cardiac troponin I (cTnI) levels over time. **(B)** Timeline of the patient's diagnosis and treatments.

The patient was treated with intravenous immunoglobulin (20 g daily, administered over a 4-h infusion period each day) for 2 days. Because the patient developed hypokalemia on the day after he had been diagnosed with acute myocarditis, we administered a combination of oral prednisolone acetate (60 mg daily), enalapril (10 mg daily), metoprolol (50 mg daily), potassium chloride sustained-release tablets (1 g daily), and broad-spectrum antibiotics for 7 days. After this treatment, the patient's symptoms improved and his cTnI levels gradually decreased, leading to hospital discharge. Approximately 1 month later, non-contrast CMR showed a clinically significant reduction in myocardial edema (T2 ratio, 1.8; T2 mapping, 42.2 ± 1.7 ms), along with a cTnI level of 0.14 μg/L. An outpatient follow-up examination after about 110 days revealed that the patient's cTnI levels had returned to baseline (Figure 4A). After improvement of myocarditis, an ECG showed abnormal Q waves and T-wave changes in leads II, III, and aVF (Figure 2B). Follow-up echocardiography findings were normal. In July 2024, enhanced CMR showed no signs of myocardial edema (T2 ratio, 1.7; T2 mapping, 42.0 ± 0.9 ms) (Figures 3C,D) but revealed an increased range of LGE in the lateral walls of the basal segment (Figures 1G,H). The patient is currently asymptomatic and continues to undergo monitoring via outpatient and telephone follow-up. The patient's timeline is summarized in Figure 4B. Written informed consent was obtained from the patient for publication of potentially identifiable images or data included in this article.

## 3 Discussion

We have presented a rare case of acute myocarditis in a patient with DMD, potentially due to upper respiratory tract infection; the condition was accurately diagnosed by CMR and successfully treated. Considering our experience in this case, we believe that there is a compelling need for further investigation into this condition. Here, we summarize the available literature.

Among the few reported cases of acute myocarditis in a patient with DMD (8–11), most patients were hospitalized due to chest pain. Our patient only presented with seemingly mild symptoms of a respiratory infection, similar to those typically observed in children. These symptoms might be easily overlooked by clinicians, especially during seasons with a high incidence of respiratory infections in children. The diagnosis of masked myocarditis in such cases should be based on a combination of laboratory findings, ECG results, and imaging reports.

Previous reports of myocarditis in patients with DMD have also noted a clinically significant increase in cardiac troponin (8, 9, 12), consistent with our findings. However, in contrast to the ECG characteristics reported in prior cases (8, 9, 11, 12), our patient exhibited abnormal Q waves in the inferior wall, in addition to ST elevations. Abnormal Q waves on ECG have been associated with worse prognosis in patients with pediatric myocarditis (13). Despite prompt treatment and close monitoring, the abnormal Q waves and T-wave changes in the inferior wall persisted, potentially indicating ongoing chronic myocardial damage. As a readily available and low-cost clinical tool, continued ECG assessment is essential for disease monitoring during treatment.

Echocardiography is the most common noninvasive method for assessing cardiac function. Our patient had pre-existing myocardial fibrosis but normal cardiac function upon admission. These findings are consistent with the results of previous studies, which suggested that myocardial fibrosis progression is not associated with left ventricular dysfunction (14–17). Some case reports (8, 9) indicated that DMD patients with myocarditis had significant left ventricular dysfunction (LVEF, 46%). However, our patient's left ventricular function was normal at the onset of myocarditis. This discrepancy may be attributed to the regular use of steroids and cardiac medications before admission. The discrepancy also implies that masked myocardial injury can precede a decline in cardiac function, highlighting the limitations of echocardiography for follow-up screening of DMD patients.

CMR, the gold standard for noninvasive diagnosis of myocarditis, is essential for confirming a diagnosis of myocarditis when cardiac troponin levels or ECG findings are abnormal. In the few reported cases of DMD-associated myocarditis, patients often presented with edema in the left ventricular free wall and corresponding LGE on CMR (8, 9). Abdul et al. found myocardial edema and LGE in both septal and lateral walls (8), consistent with our CMR results. Notably, our patient already had left ventricular free wall LGE before the onset of myocarditis. During myocarditis, however, the extent of free wall LGE increased; we also observed transmural LGE in the inferior wall and new interventricular septal LGE. The presence of transmural and septal LGE is presumably associated with a poor prognosis (15, 18).

The progression of LGE in this case implies that myocardial inflammation accelerates the progression of DMD-associated cardiomyopathy (19). Previous research has shown that dystrophin-deficient mice with an infection exhibit more severe cardiomyopathy relative to mice with normal dystrophin (20). Our case highlights the potential for upper respiratory tract infection to induce acute myocarditis, thereby accelerating the progression of DMD-associated cardiomyopathy. Moreover, the results of a previous study suggested that DMD-associated cardiomyopathy involves acute cardiac cell damage, rather than gradual progressive loss of cardiac function (21). As patients age, recurrent episodic insults to dystrophin-deficient cardiac myocytes may strongly contribute to the progression of primary cardiomyopathy (8, 9, 12, 19, 22). In addition, its potential to accelerate chronic cardiomyopathy, myocardial inflammation may also trigger an inflammatory cytokine storm, leading to heart failure and potentially fatal outcomes, as observed in the context of severe acute respiratory syndrome coronavirus 2 (SARS-CoV-2) infection (23). Thus, early diagnosis and prompt management of acute myocardial injury (e.g., acute myocarditis) in DMD patients are crucial for efforts to delay cardiomyopathy progression and improve overall outcomes. However, the etiology of acute myocarditis in patients with DMD remains unclear. It may occur spontaneously due to an autoinflammatory response or be triggered by viral infection, physiological stress, or comorbid conditions. Further pathological examination, basic research, and animal studies are needed to elucidate the underlying mechanisms.

Endocardial biopsy is the gold standard for diagnosing myocarditis because it allows further immunohistological analysis. This approach can identify active myocarditis and provide risk stratification information for clinicians. A previous study demonstrated the accuracy of CMR in diagnosing DMD-associated myocarditis, compared with endocardial biopsy findings, as well as the accuracy of CMR in distinguishing between active and healing myocarditis (19). The study also showed that DMD patients with myocardial inflammation experienced rapid progression to heart failure. However, due to the rarity of DMD-associated myocarditis and the invasive nature of endocardial biopsy, few studies have investigated the use of this procedure in DMD patients.

DMD-associated cardiomyopathy may be delayed and improved with steroids and cardioprotective therapies (e.g., ACE inhibitors, β-blockers, and mineralocorticoid receptor antagonists) (4, 8, 24, 25). Current DMD management guidelines recommend initiating cardioprotective therapy upon detection of cardiac abnormalities at any age (4). Despite receiving steroids and cardioprotective treatment, our patient experienced respiratory infection-induced myocardial inflammation that exacerbated his myocardial damage, with the potential to become life-threatening in the absence of timely intervention. There are no standardized treatment guidelines for acute myocarditis in patients with DMD. The American Heart Association recommends intravenous immunoglobulin and corticosteroids as the most common therapies for childhood myocarditis (26). Oral pulse corticosteroid therapy (e.g., methylprednisolone) may improve outcomes in DMD patients with acute myocarditis (9), although there is insufficient evidence from randomized clinical trials.

To our knowledge, there have been few reported cases of subclinical myocarditis lacking typical clinical manifestations in patients with DMD. Our case emphasizes that, in children with DMD, even mild respiratory infection symptoms deserve clinical attention. In patients with normal cardiac function, markers of myocardial injury and ECG abnormalities may provide valuable information. Further evaluation with CMR is essential to confirm a diagnosis of myocarditis. Our case also highlights the potential for upper respiratory tract infections to induce acute myocarditis, resulting in clinically significant myocardial edema and inflammation that may accelerate the progression of DMD-associated cardiomyopathy and require closer clinical follow-up. However, our study had some limitations. First, due to time constraints and emergency setting, we did not identify the specific etiology of the acute myocarditis; it may have been caused by an undetected pathogen or a spontaneous immune response, and further investigation is warranted. Second, image quality was limited due to suboptimal cooperation from the pediatric patient during examinations. Third, we did not perform endocardial biopsy at the request of the patient and his guardian.

## 4 Conclusion

This case report presents a potentially valuable diagnostic tool and effective management strategy for DMD-associated cardiomyopathy complicated by acute myocarditis. Further studies are needed to confirm the efficacy of this approach.

## Data Availability

The original contributions presented in the study are included in the article/Supplementary Material, further inquiries can be directed to the corresponding author.
